# Targeted massively parallel sequencing characterises the mutation spectrum of *PALB2* in breast and ovarian cancer cases from Poland and Ukraine

**DOI:** 10.1007/s10689-017-0050-6

**Published:** 2017-10-19

**Authors:** Aleksander Myszka, Tu Nguyen-Dumont, Pawel Karpinski, Maria M. Sasiadek, Hayane Akopyan, Fleur Hammet, Helen Tsimiklis, Daniel J. Park, Bernard J. Pope, Ryszard Slezak, Nataliya Kitsera, Aleksandra Siekierzynska, Melissa C. Southey

**Affiliations:** 1 Institute of Obstetrics and Emergency Medicine, University of Rzeszow, Rzeszow, Poland; 2 Genetic Epidemiology Laboratory, Department of Pathology, The University of Melbourne, Melbourne, Australia; 3 Precision Medicine, School of Clinical Sciences, Monash University, Clayton, Australia; 4 Department of Genetics, Wroclaw Medical University, Wroclaw, Poland; 5 Institute of Hereditary Pathology of National Academy of Medical Sciences, Lviv, Ukraine; 6 Melbourne Bioinformatics, The University of Melbourne, Melbourne, Australia; 7 Department of Biotechnology and Plant Physiology, University of Rzeszow, Rzeszow, Poland

**Keywords:** *PALB2*, Breast cancer, Ovarian cancer, Genetic susceptibility, Massively parallel sequencing

## Abstract

Loss-of-function germline mutations in the *PALB2* gene are associated with an increase of breast cancer risk. The purpose of this study was to characterise the spectrum of *PALB2* mutations in women affected with breast or ovarian cancer from South-West Poland and West Ukraine. We applied Hi-Plex, an amplicon-based enrichment method for targeted massively parallel sequencing, to screen the coding exons and proximal intron–exon junctions of *PALB2* in germline DNA from unrelated women affected with breast cancer (n = 338) and ovarian cancer (n = 89) from Poland (n = 304) and Ukraine (n = 123). These women were at high-risk of carrying a genetic predisposition to breast and/or ovarian cancer due to a family history and/or early-onset disease. Targeted-sequencing identified two frameshift deletions: *PALB2*:c.509_510del; p.R170Ifs in three women affected with breast cancer and *PALB2*:c.172_175del;p.Q60Rfs in one woman affected with ovarian cancer. A number of other previously described missense (some predicted to be damaging by PolyPhen-2 and CADD) and synonymous mutations were also identified in this population. This study is consistent with previous reports that *PALB2*:c.509_510del and *PALB2*:c.172_175del are recurrent mutations associated with breast cancer predisposition in Polish women with a family history of the disease. Our study contributes to the accumulating evidence indicating that *PALB2* should be included in genetic testing for breast cancer susceptibility in these populations to enhance risk assessment and management of women at high-risk of developing breast cancer. This data could also contribute to ongoing work that is assessing the possible association between ovarian cancer risk and *PALB2* mutations for which there is currently no evidence.

## Background

Breast cancer risk associated with carrying *PALB2* loss-of-function mutations is now well established. All published estimates of penetrance of *PALB2* mutations (recently reviewed by us [1]) are comparable to the breast cancer risk associated with mutations in *BRCA2*: 45% (95% CI, 31–56%) [2]. By pooling international resources, the *PALB2* Interest Group estimated that the average cumulative risk of breast cancer risk ranged from 33% (95% CI, 25–44%) for a female carrier without affected relatives to 58% (95% CI, 50–66%) for a female carrier with two first-degree relatives who had breast cancer diagnosed by 50 years of age [3].

There is currently no evidence for ovarian cancer risk to be associated with *PALB2* loss-of-function mutations. Recent consolidated efforts including (i) those of the *PALB2* Interest Group that estimated the relative risk for ovarian cancer to be 2.31 (95% CI, 0.77–6.97; p = 0.18), (ii) the assessment of two protein truncating mutations *PALB2*:c.1592delT; p.L531Cfs and *PALB2*:c.3113G > A;p.W1038* on iCOGS (OR 2.50, 95% CI, 0.21–29.1, p = 0.45 and OR 1.34, 95% CI, 0.36–4.97, p = 0.66, respectively) and (iii) a study of *PALB2*:c.509_510delGA and *PALB2*:c.172_175delTTGT in 344 Polish families with breast and ovarian cancer (OR 1.37, 95% CI, 0.17–10.7, p = 0.54) all failed to reach statistical significance [3–5].

This study aimed to apply massively parallel sequencing to characterise the mutation spectrum of *PALB2* in women from South-West Poland and West Ukraine affected with breast or ovarian cancer.

## Materials and methods

### Subjects

Participants in this study were unrelated women diagnosed with breast or ovarian cancer recruited after or during the oncological treatment from Wroclaw Medical University, Lower Silesia, Poland, between 2004 and 2008, or Lviv State Oncology Regional Treatment and Diagnostic Center, Lviv, Ukraine between 2004 and 2010. Genetic testing was requested when hereditary cancer was suspected (age of onset < 50, bilateral breast cancer, medullary or atypical breast cancer, more than one breast cancer in the family occurring in a first or second degree relative and ovarian cancer in any age). The time from cancer diagnosis to blood draw ranged from 1 to 12 months.

The Polish cohort consisted of 226 women affected with breast cancer and 78 women affected with ovarian cancer. Of the 226 women with breast cancer, 85 had hereditary breast cancer, 17 had familial breast cancer and 124 were sporadic cases, according to the criteria described by Berliner et al. [6]. The majority of these women (n = 206, 91%) had been diagnosed with invasive cancer (ductal in 153, lobular in 30, medullary in 7, tubular in 5 cases, 11 patients had been diagnosed with other types of carcinoma). There were 20 cases of cancer in situ (ductal carcinoma in situ—DCIS in 19, lobular carcinoma in situ—LCIS in 1). Of the 78 Polish women with ovarian cancer, 11 had hereditary ovarian cancer, 10 had familial ovarian cancer and 57 were sporadic ovarian cancer cases. Thirty-eight patients had serous cancer, 15 had endometroid, 10 had mucinous, 2 had clear cell, 13 had adenocarcinoma not otherwise specified. Known carriers of Polish founder mutations in *BRCA1* (c.5266dup, c.181T > G, c.4035del, c.68_69del) and in *BRCA2* (c.5946delT) were excluded from this study.

The Ukrainian cohort consisted of 112 women with breast cancer and 11 women with ovarian cancer. Seventy-four women affected with breast cancer were diagnosed with hereditary cancer and 38 with familial cancer. There were 78, 18 and 2 cases of invasive ductal, lobular and medullary breast cancers, respectively. Of the 11 Ukrainian women with ovarian cancer, two had moderately differentiated carcinoma, one had endometroid adenocarcinoma, one had serous papillary adenocarcinoma, one had low-grade differentiated adenocarcinoma and six had adenocarcinoma not otherwise specified. Additional information on the participants is presented in Table 1.

**Table 1:** Characteristics of the participants  of this study

	Breast cancer		Ovarian cancer	
	Poland (n = 226)	Ukraine (n = 112)	Poland (n = 78)	Ukraine (n = 11)
Age at diagnosis (years)	49 (22–72)	50 (28–79)	53 (25–80)	51 (31–65)
Relatives with breast cancer				
0	148 (65%)	24 (21%)	61 (%)	5 (45%)
1	51 (23%)	56 (50%)	14 (%)	6 (55%)
2+	27 (12%)	32 (29%)	3 (%)	0 (0%)
Relatives with ovarian cancer				
0	210 (93%)	98 (86%)	65 (%)	6 (55%)
1	13 (6%)	12 (11%)	10 (%)	3 (27%)
2+	3 (1%)	2 (2%)	3 (%)	2 (18%)
Invasive cancers				
GI	14 (11%)	NA	8 (18%)	NA
GII	65 (52%)	NA	18 (41%)	NA
GIII	46 (37%)	NA	18 (41%)	NA
In situ cancers				
GI	1 (20%)	NA	–	–
GII	2 (40%)	NA	–	–
GIII	2 (40%)	NA	–	–

*NA* data not available, *GI* grade I, *GII* grade II, *GIII* Grade III

All participants provided informed consent for participation in this research program, which was approved by the Commission of Bioethics of the Institute of Hereditary Pathology of the National Academy of Medical Sciences of Ukraine, the Ethics Committee of Wroclaw Medical University (Poland), the Ethics Committee of University of Rzeszow (Poland) and the University of Melbourne Human Research Ethics Committee (Melbourne, Australia).

### Mutation screening

Amplicon-based massively parallel sequencing of the coding regions and proximal intron–exon junctions of *PALB2* (NM_024675.3) was performed on lymphocytes-derived genomic DNA using the Hi-Plex protocol [7]. Massively parallel sequencing (150 bp paired-end) was performed on the MiSeq (Illumina, San Diego, CA, USA). Mapping to human reference build hg19 and variant calling were performed as described in [7, 8].

### In-silico analysis

DNA sequence variant annotation (variant nomenclature and type, and dbSNP138 identifier) was performed using CAVA [9]. The probability that missense substitutions in *PALB2* were damaging to protein function was assessed with PolyPhen-2 [10] and CADD [11]. The threshold for calling a missense variant damaging was the default for PolyPhen-2. The cutoff for CADD was 15, as recommended by the authors. Minor Allele Frequency (MAF) was obtained for non-Finnish European ancestry from the ExAC database [12].

## Results

A total of 23 distinct *PALB2* genetic variants were observed in the DNA from 427 women (Table 2). Two frameshift deletions resulting in predicted premature termination codons were identified: *PALB2*:c.509_510del; p.R170Ifs in three women affected with breast cancer and *PALB2*:c.172_175del;p.Q60Rfs in one woman affected with ovarian cancer.

**Table 2:** *PALB2* variants identified by Hi-Plex targeted-sequencing, in 433 women affected with breast or ovarian cancer in South-West Poland and West Ukraine

	HGVS_c^a^	HGVS_p^a^	dbSNP^b^	MAF in ExAC^c^	PolyPhen-2^d^	CADD^e^	# Carriers	
							BC^f^	OC^g^
Frameshifting deletions	c.172_175del	p.Q60Rfs*7	–	–	–	–	0	1
	c.509_510del	p.R170Ifs*14	–	0.00010	–	–	3	0
Missense substitutions	c.13C > T	p.P5S	rs377085677	0.00005	B	13.2	3	0
	c.187C > G	p.L63V	–	0.00001	B	0.014	1	0
	c.1010T > C	p.L337S	rs45494092	0.01920	B	8.918	6	3
	c.1033T > G	p.L345V	–	–	B	3.477	1	0
	c.1544A > G	p.K515R	–	0.00006	B	16.16	1	0
	c.1676A > G	p.Q559R	rs152451	0.09550	B	0.029	75	17
	c.2014G > C	p.E672Q	rs45532440	0.02830	B	11.43	24	5
	c.2135C > T	p.A712V	rs141458731	0.00040	B	12.2	0	1
	c.2590C > T	p.P864S	rs45568339	0.00410	B	12.03	1	1
	c.2773G > C	p.V925L	rs180177125	0.00005	B	14.83	1	0
	c.2794G > A	p.V932M	rs45624036	0.00810	D	18.31	4	2
	c.2816T > G	p.L939W^h^	rs45478192	0.00160	D	20.9	1	1
	c.2993G > A	p.G998E	rs45551636	0.02140	D	22.7	15	3
	c.3508C > T	p.H1170Y	rs200283306	0.00010	D	16.95	2	0
Synonymous substitutions	c.615A > G	p.E205E	–	0.00003	–	–	1	0
	c.1194G > A	p.V398V	rs61755173	0.00120	–	–	1	1
	c.1281T > C	p.A427A	rs138697796	0.00000	–	–	1	0
	c.1572A > G	p.S524S	rs45472400	0.00410	–	–	1	0
	c.1968A > G	p.P656P	–	–	–	–	1	0
	c.3252G > A	p.S1084S	rs141570833	0.00004	–	–	1	0
	c.3300T > G	p.T1100T	rs45516100	0.02830	–	–	23	5

^a^Variant nomenclature based on transcript sequence (NM_024675.3), + 1 as A of ATG start codon, according to the Human Genome Variation Society (HGVS), HGVS_c for coding DNA and HGVS_p for protein variants.

^b^dbSNP 138

^c^Minor Allele Frequency (MAF) in ExAC Non-Finnish European population [12]

^d^PolyPhen-2 prediction: B, benign; D: damaging [10]

^e^CADD score [11]

^f^ *BC* breast cancer

^g^ *OC* ovarian cancer

^h^No evidence for association with breast or ovarian cancer risk [4]

Of the 14 missense substitutions identified, four were predicted to be damaging by both PolyPhen-2 and CADD. The remaining variants were synonymous variants. No nonsense mutation or variant affecting consensus splice sites were detected.

Two Polish women were identified as carriers of *PALB2*:c.509_510del. One of them had been diagnosed with invasive lobular breast carcinoma at 35 years of age. There was no other case of cancer reported in her family. The second Polish carrier of *PALB2*:c.509_510del had been diagnosed with mucinous carcinoma of the breast at 36 years of age. Other cancers in her family included her paternal grand-mother and aunt (breast cancer, unknown age of diagnoses) and cancer of the larynx in her father. The third carrier of *PALB2*:c.509_510del was diagnosed at 53 years with infiltrating ductal carcinoma. This woman’s mother had also been diagnosed with breast cancer at an unknown age.

The carrier of *PALB2*:c.172_175del was a Polish woman diagnosed with papillary serous ovarian cancer at the age of 57 years. She had a family history of cancer that included lung cancer (brother diagnosed at 36 years), liver cancer (sister diagnosed at 60 years), mouth cancer (mother diagnosed at 69 years) and gastric cancer (maternal uncle diagnosed at 48 years).

## Discussion–conclusion

Both frameshift deletions observed in this study have been reported previously to be recurrent in the Polish population. *PALB2*:c.509_510del was first identified by Dansonka-Mieszkowska et al. in a study of women with breast or ovarian cancer from South Poland [13]. They observed the deletion in 4/648 (0.6%) familial breast cancer cases and 1/1310 (0.08%) controls (p = 0.044). *PALB2*:c.172_175del was initially reported as a Czech Republic founder mutation (4/409 high-risk breast cancer cases) [14]. Further studies have confirmed that *PALB2*:c.509_510del and *PALB2*:c.172_175del are recurrent in the Polish population [15–17]. Cybulski et al. [17] estimated the odds ratios for risk of breast cancer for *PALB2*:c.509_510del carriers and *PALB2*:c.172_175del carriers to be 4.09 (95% CI 1·89–8·88; p < 0·0001) and 5.02 (1·55–16·2; p = 0·0016), respectively, consistent with breast cancer risk estimates obtained for *PALB2*:c.1592del;p.L531Cfs and *PALB2*:c.3113G > A;p.W1038* [4]. Ten-year survival for women with breast cancer and a *PALB2* mutation was 48.0% (95% CI, 36.5–63.2%), compared with 74.7% (95% CI, 73.5–75.8%) for non-carriers (hazard ratio for death 2.27, 95% CI, 1.64–3.15; p < 0.0001) [5].

Most of the previous studies have applied genotyping to detect these deletions, except for [17], where whole-exome sequencing was performed on 144 women and identified three carriers. The present study is the first to apply massively parallel sequencing to assess the full spectrum of exonic and consensus splice site genetic variants in *PALB2* in the population of South-West Poland and West Ukraine.

Our findings further support that *PALB2* should be included in genetic testing for breast cancer susceptibility in these populations. For women who meet criteria for genetic testing in Poland, testing for the known Polish founder mutations in *PALB2*, together with Polish founder *BRCA1* mutations could be recommended. For those found negative, gene-panel testing by massively parallel sequencing (next-generation sequencing) could be applied to facilitate risk assessment and management for these women at high-risk of developing breast cancer.

Interpretation of the rare genetic variation observed in *PALB2*, especially the rare missense variants, is challenging. As discussed by us and others, there has been no evidence so far that rare missense variants in *PALB2* are associated with increased risk of breast cancer [1, 18, 19]. The inclusion of *PALB2* on gene-panel tests for breast cancer susceptibility will contribute to the accumulation of data that could be used to estimate cancer risks associated with missense variants in *PALB2* (and other genes included on these panels), on a variant-by-variant basis.
